# Targeting cholesterol homeostasis in lung diseases

**DOI:** 10.1038/s41598-017-10879-w

**Published:** 2017-08-31

**Authors:** Anthony Sallese, Takuji Suzuki, Cormac McCarthy, James Bridges, Alyssa Filuta, Paritha Arumugam, Kenjiro Shima, Yan Ma, Matthew Wessendarp, Diane Black, Claudia Chalk, Brenna Carey, Bruce C. Trapnell

**Affiliations:** 1 Translational Pulmonary Science Center, Children’s Hospital Medical Center, Cincinnati, OH USA; 2 Division of Pulmonary Biology, Children’s Hospital Medical Center, Cincinnati, OH USA; 3 Graduate Program in Pathobiology and Molecular Medicine, University of Cincinnati College of Medicine, Cincinnati, OH USA; 4 Division of Pulmonary Medicine, Children’s Hospital Medical Center, Cincinnati, OH USA; 5 Division of Pulmonary, Critical Care, and Sleep Medicine, University of Cincinnati College of Medicine, Cincinnati, OH USA

## Abstract

Macrophages are critical to organ structure and function in health and disease. To determine mechanisms by which granulocyte/macrophage-colony stimulating factor (GM-CSF) signaling normally maintains surfactant homeostasis and how its disruption causes pulmonary alveolar proteinosis (PAP), we evaluated lipid composition in alveolar macrophages and lung surfactant, macrophage-mediated surfactant clearance kinetics/dynamics, and cholesterol-targeted pharmacotherapy of PAP *in vitro* and *in vivo*. Without GM-CSF signaling, surfactant-exposed macrophages massively accumulated cholesterol ester-rich lipid-droplets and surfactant had an increased proportion of cholesterol. GM-CSF regulated cholesterol clearance in macrophages in constitutive, dose-dependent, and reversible fashion but did not affect phospholipid clearance. PPARγ-agonist therapy increased cholesterol clearance in macrophages and reduced disease severity in PAP mice. Results demonstrate that GM-CSF is required for cholesterol clearance in macrophages, identify reduced cholesterol clearance as the primary macrophage defect driving PAP pathogenesis, and support the feasibility of translating pioglitazone as a novel pharmacotherapy of PAP.

## Introduction

GM-CSF has emerged as an important regulator of the ontogeny, renewal, and functions of macrophages in health and disease, particularly in the lung^1–4^. For example, pulmonary alveolar macrophages require GM-CSF to maintain surfactant homeostasis, which is critical to alveolar stability and lung function^5, 6^, and disruption of GM-CSF signaling causes pulmonary alveolar proteinosis (PAP) – a syndrome of progressive alveolar surfactant accumulation and resulting hypoxemic respiratory failure that occurs in men, women and children^3, 7^. In humans, PAP is caused by neutralizing GM-CSF autoantibodies^8, 9^ or mutations in *CSF2RA* ^10, 11^ or *CSF2RB* ^12, 13^ encoding GM-CSF receptor α and β, respectively. In mice, PAP develops after gene ablation of *Csf2* ^5, 6^ or *Csf2rb* ^14^. In each, the lung disease is histologically, biochemically, and physiologically similar and driven by disruption of GM-CSF signaling, which alveolar macrophages require to clear surfactant normally^15^. However, the mechanism responsible is not known.

Surfactant is composed of 80% polar lipids (primarily saturated phosphatidylcholine (SatPC) and other less-abundant phospholipids), 10% neutral lipids (primarily free cholesterol with small amounts of triglycerides and free fatty acids), and 10% proteins^16, 17^. Cholesterol content regulates surfactant fluidity and function in lunged animals and can change rapidly, especially under extremes of temperature^18^. Surfactant homeostasis is maintained by balanced secretion by alveolar epithelial type II cells and clearance via recycling and catabolism in these cells and catabolism in alveolar macrophages^19^. Reports that the relative composition of surfactant phospholipids is normal in PAP patients^20^ and *Csf2*−/− mice^21, 22^ led to a widely-held belief that surfactant accumulation in PAP is caused by impaired catabolism of phospholipids in alveolar macrophages^15^, however, no such mechanism has been found.

The observation that in PAP, alveolar macrophages have abnormal expression of PPARγ^23–25^, ABCA1^25, 26^ and ABCG1^23^, factors important in cholesterol transport in macrophages^26^, suggested the hypothesis that disruption of cholesterol homeostasis rather than impaired surfactant phospholipid catabolism in macrophages may drive pathogenesis. We addressed this hypothesis by first identifying the lipids accumulating within alveolar macrophages in PAP. We also evaluated GM-CSF regulation of surfactant uptake and clearance by macrophages, and PPARγ- or LXR-agonist mediated pharmacologic correction of PAP-related abnormalities in murine macrophages *in vitro* and *in vivo*.

## Lipid Accumulation in PAP Macrophages

**Figure 1:**
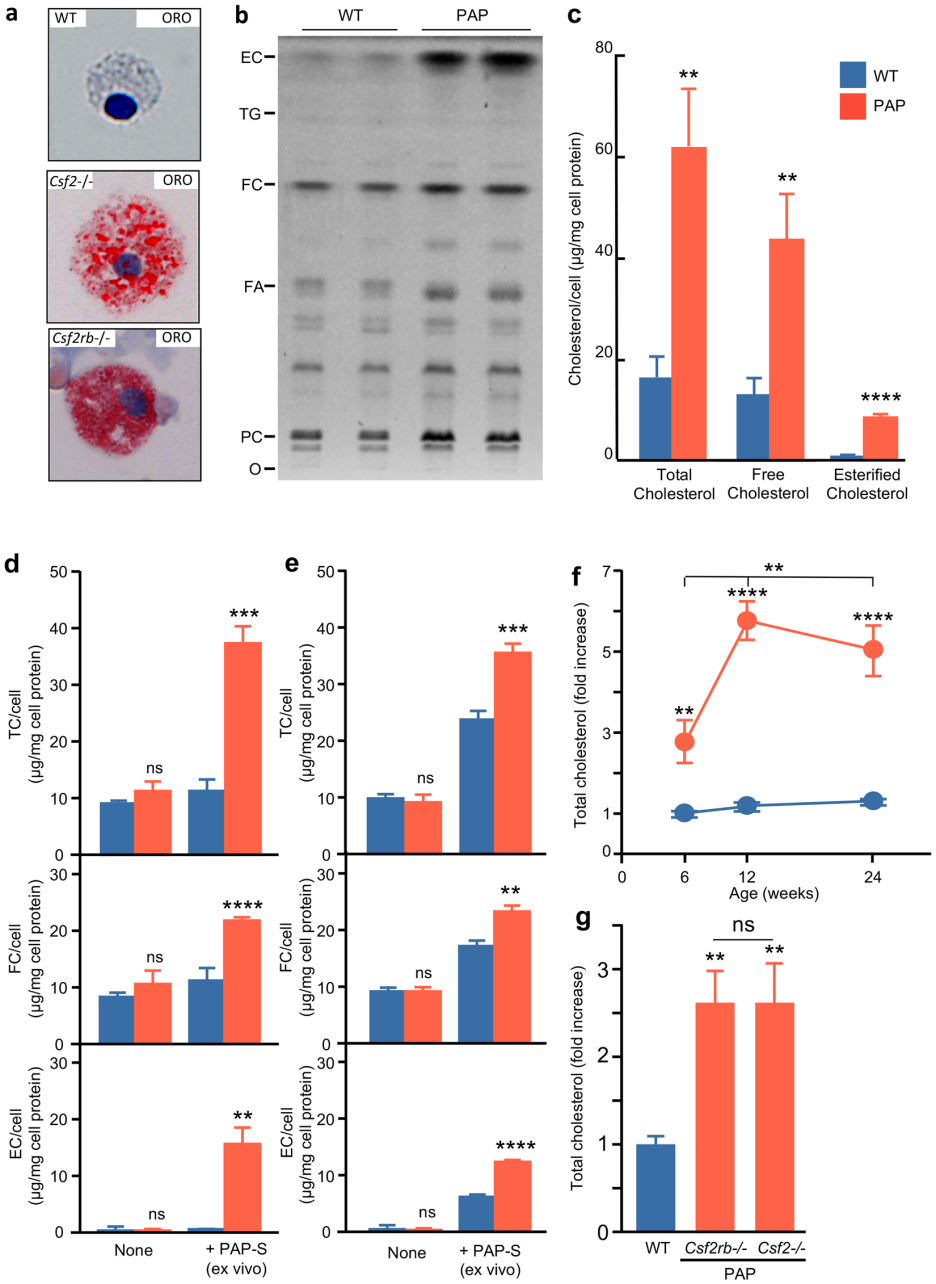
Cholesterol accumulation in surfactant-exposed macrophages. (**a**) Alveolar macrophages from 6 week-old WT or PAP mice (*Csf2*−/−, or *Csf2rb*−/−) stained with oil-red-O (ORO) demonstrating neutral lipid accumulation (EM). (**b**) TLC/primuline staining of alveolar macrophage total lipid extracts. Markers: esterified cholesterol (EC), triglycerides (TG), free cholesterol (FC), fatty acids (FA), phosphatidylcholine (PC), origin (O), each lane consists of alveolar macrophage lipids from 1 WT mice or 2 pooled *Csf2*−/− mice. (**c**) Levels of total, free, and esterified cholesterol (TC, FC, EC) from alveolar macrophages of 6 week-old WT or PAP mice. (**d**) Levels of total, free, and esterified cholesterol (TC, FC, EC) from BMD macrophages without (none) or with surfactant exposure (+PAP-S). (**e**) Levels of total, free, and esterified cholesterol (TC, FC, EC) from peritoneal macrophages without (none) or with surfactant exposure (+PAP-S). (**f**) Alveolar macrophage total cholesterol level in WT or PAP mice at 6, 12 and 24 weeks of age. (**g**) Alveolar macrophage cholesterol content from WT, *Csf2*−/− *and Csf2rb*−/− mice. Data are mean ± SD of 3 mice per group. *P < 0.05, **P < 0.01, ***P < 0.001, ****P < 0.0001, not significant (ns).

We characterized the lipids accumulating in alveolar macrophages in PAP since the earliest cellular abnormality following disruption of GM-CSF signaling is the development of foamy alveolar macrophages enlarged by accumulation of intracytoplasmic lipid droplets^9^. Alveolar macrophages from mice with PAP stained positive with oil-red-O indicating the presence of neutral lipids; fatty acids, triglycerides or cholesterol ester^27, 28^ (Fig. 1a). Surfactant lipid composition was evaluated relative to total surfactant lipids rather than to the polar lipid fraction as prior studies had done^21^ using a novel thin layer chromatography (TLC) method permitting simultaneous evaluation of polar and non-polar lipids. Compared to WT mice, alveolar macrophages from *Csf2*−/− mice had increased levels of esterified and free cholesterol but no major changes in triglycerides or free fatty acids, and minimal changes in levels of phospholipid species (Fig. 1b). Using a quantitative fluorometric method, we found that, compared to corresponding WT controls, total, free, and particularly esterified cholesterol levels were markedly increased in alveolar macrophages from *Csf2*−/− mice (Fig. 1c).

To determine if this phenotype was specific to alveolar macrophages or common to other macrophage populations, similar studies were done with bone marrow derived (BMD) and peritoneal macrophages. Compared to WT controls, BMD macrophages from *Csf2*−/− mice had no increase in cholesterol levels at baseline but had marked increases in both free and esterified cholesterol after exposure to PAP patient-derived surfactant (PAP-surfactant) (Fig. 1d). Similar results were seen for peritoneal macrophages at baseline and after exposure to PAP-surfactant (Fig. 1e). Compared to age-matched WT controls, the total cholesterol level in alveolar macrophages from *Csf2*−/− mice was increased 2.9-fold at six weeks, rose further to 5.4-fold at 12 weeks, plateaued at 5-fold at 24 weeks (Fig. 1f), and was similarly increased in both *Csf2*−/− and *Csf2rb*−/− mice (Fig. 1g). These results indicate that esterified and free cholesterol are the predominant lipid species accumulating in alveolar macrophages in PAP mice, GM-CSF regulation of cholesterol clearance is not limited to alveolar macrophages but is common to other macrophage populations, and accumulation of cholesterol is not an intrinsic defect caused by GM-CSF deficiency but requires exposure to surfactant.

## GM-CSF Regulates Surfactant Composition

**Figure 2:**
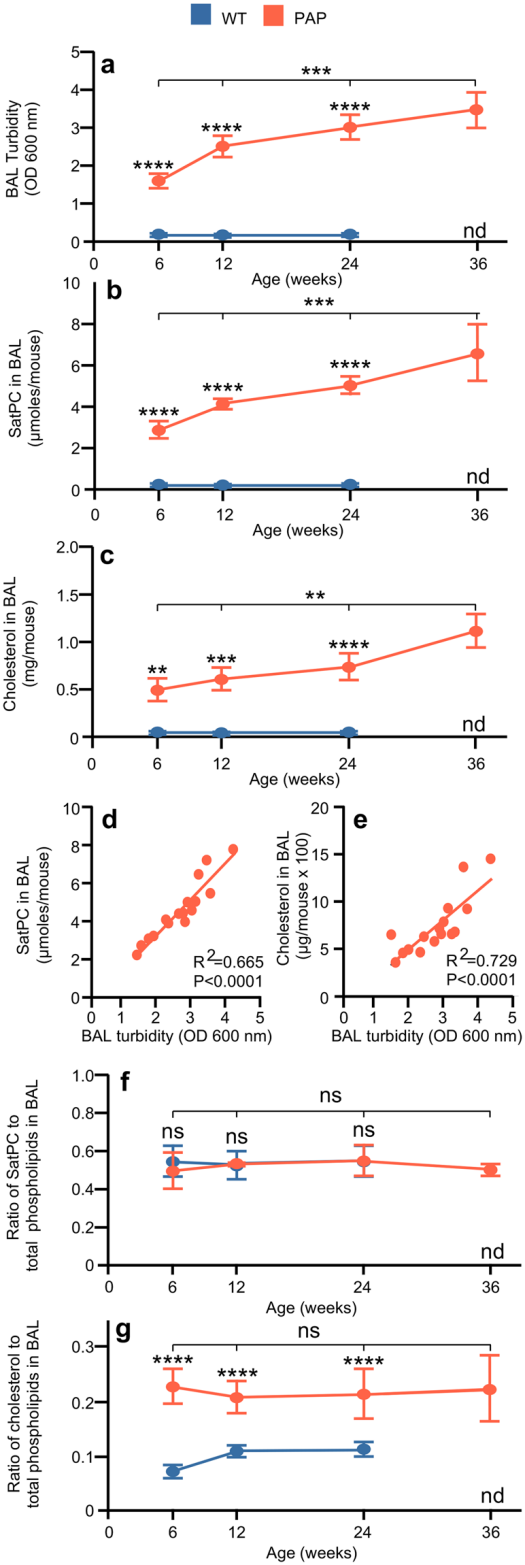
Changes in surfactant lipid composition in PAP mice. (**a**) Bronchoalveolar lavage (BAL) turbidity (optical density (OD) at λ = 600 nm). (**b**) Saturated phosphatidylcholine (SatPC) levels in BAL from WT and PAP mice. (**c**) Cholesterol levels in BAL from WT and PAP mice. (**d**) Correlation between SatPC levels in BAL and turbidity. (**e**) Correlation between cholesterol levels in BAL and turbidity. (**f**) Ratio of SatPC to total phospholipids in BAL from WT and PAP mice. (**g**) Ratio of cholesterol to total phospholipids in BAL from WT and PAP mice. Data are mean ± SD of 4 (mice) per group or time-point. *P < 0.05, **P < 0.01, ***P < 0.001, ****P < 0.0001. Not done (nd).

We next sought to determine if disruption of GM-CSF signaling alters surfactant lipid composition or if surfactant lipid composition changes with disease progression as measured by bronchoalveolar lavage (BAL) turbidity, an excellent global measure of surfactant accumulation^4^. BAL turbidity was increased with age in *Csf2*−/− mice while remaining low and constant in WT mice (Fig. 2a). Both SatPC and cholesterol in BAL were increased in surfactant from *Csf2*−/− mice compared to WT mice (Fig. 2b,c) and BAL turbidity correlated well with age-dependent, disease severity-related changes in both SatPC and cholesterol (Fig. 2d,e). The ratio of SatPC to total phospholipids in surfactant from *Csf2*−/− mice was similar to WT mice and did not change with age (Fig. 2f), consistent with the concept that the relative composition of surfactant phospholipid species is not altered in PAP^21^. Importantly, the ratio of cholesterol to total phospholipids in surfactant was already elevated 3-fold in *Csf2*−/− mice at 6 weeks compared to WT, and remained elevated but did not increase further at 12, 24, and 36 weeks (Fig. 2g), whereas PAP lung disease severity increased steadily (Fig. 2a). In summary, while the relative composition of surfactant phospholipids was not altered in *Csf2*−/− mice, the relative proportion of cholesterol in surfactant was increased.

## Cholesterol Drives Lipid Accumulation

**Figure 3:**
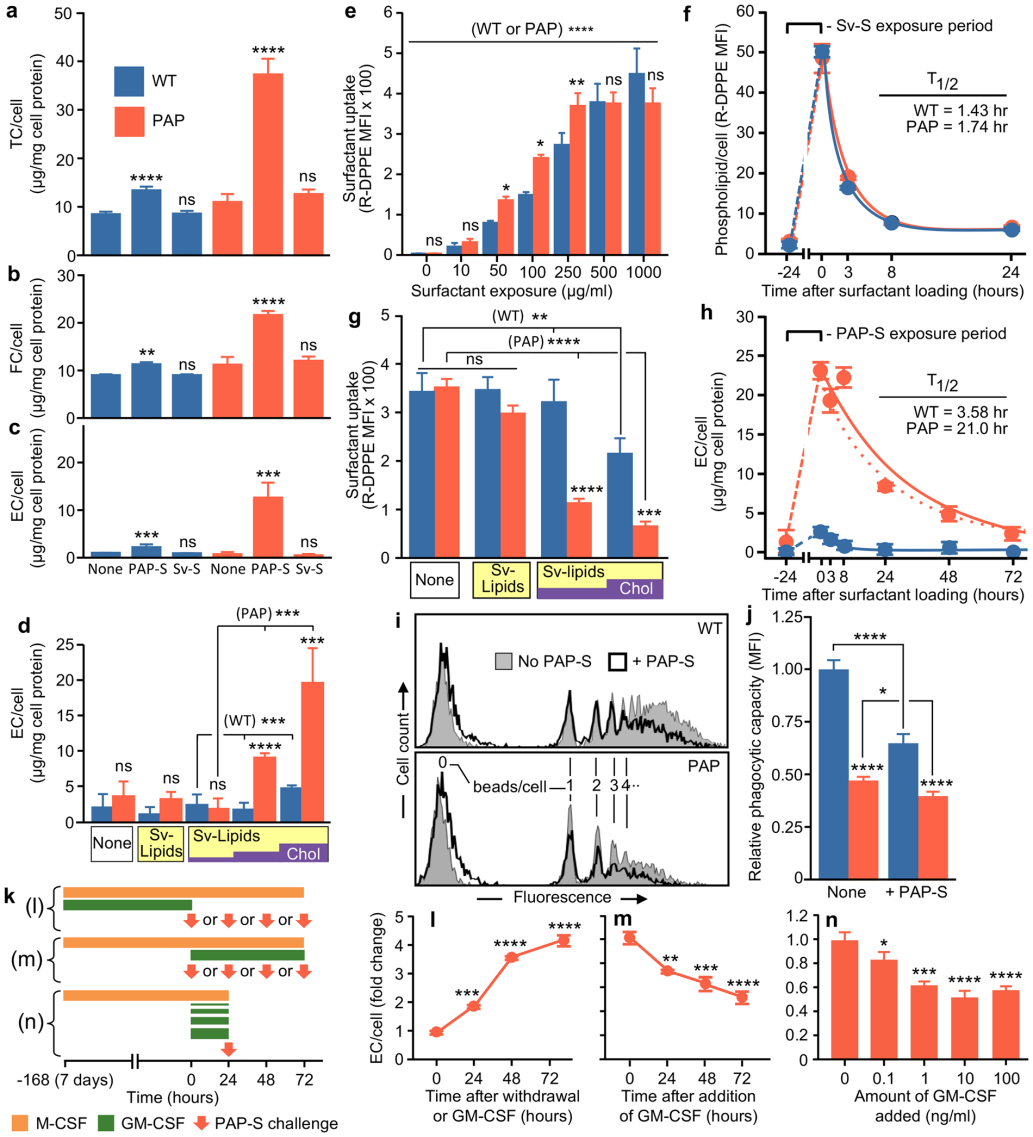
Cholesterol drives lipid accumulation in PAP macrophages. (**a**–**d**) Total, free and esterified cholesterol (TC, FC, EC)levels in BMD macrophages without or after exposure to PAP-patient derived surfactant (PAP-S), (Survanta surfactant) Sv-S, cholesterol-free Survanta lipid extract Sv-S Lipids, or Sv-S Lipids supplemented with cholesterol 10%, 25%, or 50% (wt/wt) (Sv-Lipids/Chol) evaluated as described above (Fig. 1f,g). (**e**,**f**) Uptake (**e**) and clearance (**f**) of Sv-S spiked with rhodamine-conjugated dipalmitoylphosphatidyl-ethanolamine (R-DPPE) by BMD macrophages determined by flow cytometry. (**g**) Uptake of R-DPPE-spiked Sv-S by BMD macrophages without (None) or 24 hours after exposure to Sv-Lipids or Sv-Lipids supplemented with 25% or 50% free cholesterol (wt/wt) (Sv-Lipids/Chol) determined by flow cytometry. (**h**) Cholesterol clearance kinetics after pulse exposure to PAP-S for 24 hours, cell washing, and measurement of EC/cell. (**i**,**j**) Phagocytosis of opsonized, fluorescent microspheres by BMD macrophages without and after exposure to PAP-S for 24 hours determined by flow cytometry and measurement of mean fluorescence intensity (MFI). (**k**–**n**) Kinetics/dynamics of GM-CSF-regulated cholesterol clearance. (**k**) Schematic showing times BMD macrophages were exposed to M-CSF/GM-CSF and taken to initiate exposure to PAP-S (arrows) and measurement of EC accumulation as described in Methods. Loss (**l**), restoration (**m**), and concentration-dependent stimulation (**n**) of cholesterol clearance by GM-CSF. Data are mean ± SD of 3 determinations/condition. *P < 0.05, **P < 0.01, ***P < 0.001, ****P < 0.0001.

To determine if surfactant lipid(s) may contribute to disruption of macrophage homeostasis, BMD macrophages from WT or *Csf2*−/− mice were exposed to PAP-surfactant or phospholipid-containing/cholesterol-free pharmaceutical surfactant (Survanta-surfactant) and then accumulation of cholesterol in macrophages was measured by a fluorometric method. PAP-surfactant exposure caused a marked increase in the levels of total, free, and, especially, esterified cholesterol in *Csf2*−/− macrophages and a smaller but highly significant increase in WT macrophages (Fig. 3a–c). In contrast, Survanta-surfactant exposure did not cause accumulation of either free or esterified cholesterol in either *Csf2*−/− or WT macrophages (Fig. 3a–c). To confirm this observation, BMD macrophages from WT and *Csf2*−/− mice were evaluated with or without exposure to either purified cholesterol-free Survanta-surfactant lipid extract (Survanta-lipids), or purified Survanta-lipids supplemented with free cholesterol. Exposure to Survanta-lipids did not cause cholesterol accumulation in either WT or *Csf2*−/− macrophages (Fig. 3d). In contrast, exposure to purified Survanta-lipids plus cholesterol caused marked accumulation of cholesterol in proportion to the fractional cholesterol exposure level in *Csf2*−/− macrophages and a smaller but significant degree of accumulation in WT macrophages (Fig. 3d). These results demonstrate that exposure to cholesterol but not to surfactant phospholipids caused cholesterol to accumulate in macrophages in the absence of GM-CSF signaling and to a lesser but significant level in WT macrophages, albeit, at a higher exposure level.

## GM-CSF Regulates Cholesterol Clearance

To determine how GM-CSF regulates clearance of surfactant phospholipids and cholesterol in macrophages, we first examined the kinetics of *in vitro* uptake and clearance of Survanta-surfactant spiked with rhodamine-labeled phosphatidylethanolamine by BMD macrophages from WT and *Csf2*−/− mice. Uptake of labeled Survanta-surfactant was similar in WT and *Csf2*−/− macrophages over a wide range of exposure levels as shown by the mean fluorescence intensity (MFI) of cells immediately after brief exposure (Fig. 3e). Clearance of labeled Survanta-surfactant was also similar in WT and *Csf2*−/− macrophages as shown by values for the half-life of phospholipid clearance: 1.43 and 1.74 hours, respectively (Fig. 3f). In contrast, pre-exposure to purified Survanta-surfactant lipids supplemented with cholesterol resulted in marked reduction (up to 80.5 ± 2.1%) in subsequent uptake of labeled Survanta-surfactant by *Csf2*−/− macrophages in proportion to the fractional cholesterol content during prior exposure and a smaller but significant reduction by WT macrophages (Fig. 3g). Further, the half-life of cholesterol clearance was markedly increased in PAP-surfactant-exposed *Csf2*−/− macrophages compared to WT macrophages: half-life = 21 hours versus 3.58 hours, respectively (Fig. 3h). These results indicate that disruption of GM-CSF signaling does not impair the intrinsic capacity of macrophages to metabolize surfactant phospholipids, i.e. there is no major primary (intrinsic) defect in surfactant phospholipid catabolism in *Csf2*−/− macrophages. Rather, disruption of GM-CSF signaling causes a primary macrophage defect in cholesterol clearance that results in a secondary reduction in both uptake and clearance of surfactant. Exposure to PAP-surfactant also impaired phagocytosis by BMD macrophages from both WT and *Csf2rb*−/− mice (Fig. 3i,j).

To further define the mechanism by which GM-CSF regulates cholesterol clearance, BMD macrophages were first cultured in M-CSF with (or without) GM-CSF and then GM-CSF was removed (or added-back) for various times (or at various concentrations) followed by exposure to PAP-surfactant and then measurement of intracellular cholesterol accumulation (Fig. 3k). Withdrawal of GM-CSF reduced the capacity of macrophages to clear cholesterol in a time-dependent way, which manifested as an 86% increase in exposure-related cholesterol ester accumulation by 24 hours that worsened to a 309% increase by 72 hours (Fig. 3l). Addition of GM-CSF to macrophages previously cultured without it increased the capacity of macrophages to clear cholesterol in a time-dependent way, which manifested as a 27% reduction in exposure-related cholesterol ester accumulation by 24 hours that improved to a 49% reduction by 72 hours (Fig. 3m). Further, GM-CSF increased the cholesterol clearance capacity of macrophages in dose-dependent fashion (Fig. 3n). These results indicate that regulation of cholesterol clearance in macrophages by GM-CSF is constitutive, reversible and concentration-dependent.

## Cholesterol Homeostatic Pathway Regulation

**Figure 4:**
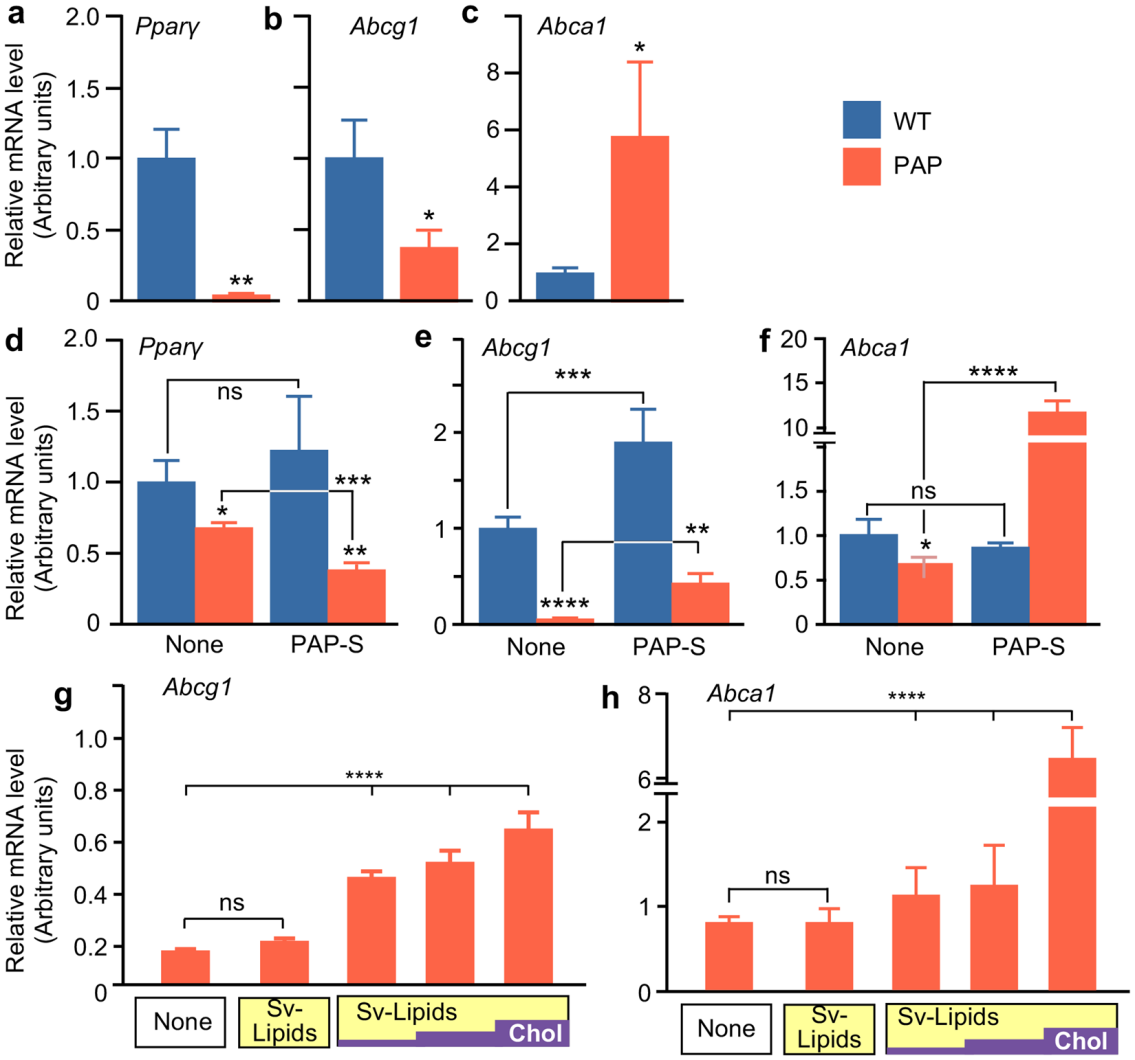
Expression of cholesterol transporter genes in PAP macrophages. (**a**–**c**) mRNA levels of Pparγ Abcg1 and Abca1 in primary alveolar macrophages from the WT and PAP mice determined by RT-PCR. (**d**–**h**) mRNA levels of Pparγ Abcg1 and Abca1 in cultured BMD macrophages (measured by RT-PCR) without or 24 hours exposure to PAP-patient derived surfactant (PAP-S) (**d**–**f**) or cholesterol-free Survanta lipid extract (Sv-Lipids) or Sv-Lipids supplemented with cholesterol to 10%, 25% or 50% (wt/wt) as indicated by yellow/purple bars (Sv-Lipids/Chol) (**g**–**h**). Data are mean ± SD (3 separate determinations/condition). *P < 0.05, **P < 0.01, ***P < 0.001, ****P < 0.0001.

The relative expression of *Pparγ* mRNA was markedly reduced in alveolar macrophages from *Csf2*−/− mice compared to WT mice (Fig. 4a) as was *Abcg1* mRNA (Fig. 4b). In contrast, *Abca1* mRNA was increased in *Csf2*−/− mice (Fig. 4c) in agreement with a previous report^23^. Alveolar macrophages from *Csf2*−/− mice also had reduced mRNA for several factors important in regulation of cholesterol homeostasis including *Lxrα* (liver X receptor alpha), *Acat1* (cholesterol acetyltransferase 1), *Nceh1* (neutral cholesterol ester hydrolase) and *Lipa* (lysosomal acid lipase) (Supplementary Fig. 1a–d). Because ABCA1 and ABCG1 are involved in cholesterol clearance in macrophages and expression of both is abnormal in alveolar macrophages in humans and mice with PAP caused by disruption of GM-CSF mediated PPARγ signaling, we determined if the changes in expression were primary (i.e., due to the loss of GM-CSF signaling) or secondary (i.e., due to exposure to surfactant). BMD macrophages from WT and *Csf2*−/− mice were exposed to PAP-surfactant or purified Survanta-surfactant lipids with or without added cholesterol. *Pparγ* mRNA levels were reduced in *Csf2*−/− macrophages compared to WT controls at baseline (Fig. 4d, left) and following exposure to PAP-surfactant were reduced further (Fig. 4d, right). *Abcg1* mRNA was severely reduced in *Csf2*−/− macrophages compared to WT controls at baseline (Fig. 4e, left). After exposure to PAP-surfactant, *Abcg1* mRNA was increased to higher than normal in WT macrophages and significantly increased in *Csf2*−/− macrophages, albeit still below normal (Fig. 4e, right). *Abca1* mRNA levels were reduced in *Csf2*−/− macrophages compared to WT controls at baseline (Fig. 4f, left). After exposure to PAP-surfactant, *Abca1* mRNA was markedly increased in *Csf2*−/− macrophages and unchanged in WT macrophages (Fig. 4f, right). To confirm these findings, BMD macrophages from *Csf2*−/− mice were exposed to purified Survanta-lipids with or without cholesterol supplementation. Exposure to purified, cholesterol-free Survanta-lipids did not alter *Abcg1* or *Abca1* mRNA levels in macrophages (Fig. 4g,h). In contrast, exposure to purified Survanta-surfactant lipids supplemented with cholesterol increased the level of *Abcg1* mRNA (Fig. 4g) in proportion to the cholesterol content and also increased *Abca1* mRNA to a smaller but significant degree (Fig. 4h). These results indicate that disruption of GM-CSF signaling caused a primary reduction in both *Abca1* and *Abcg1* mRNA in macrophages and that subsequent exposure to cholesterol-containing surfactant, but not cholesterol-free surfactant phospholipids, secondarily increased expression of both; resulting in higher-than-normal levels of *Abca1* but only partial restoration of *Abcg1* mRNA. GM-CSF signaling disruption also reduced alveolar macrophage mRNA for several factors critical to cholesterol homeostasis including *Lxrα*, *Acat1*, *Nceh1*, and *Lipa* (Supplementary Fig. 1). Since both NCEH1 and LIPA de-esterify cholesterol-esters^29–31^, a rate limiting step in cholesterol efflux^32, 33^, further studies are needed to determine the precise mechanism responsible for cholesterol ester accumulation.

## Cholesterol Homeostasis as a Novel Therapeutic Target

Because PPARγ is a master transcriptional regulator of alveolar macrophage phenotype specification^34, 35^ and stimulates expression of both ABCA1 and ABCG1, molecules important in cholesterol export from macrophages^26, 36^, we tested the hypothesis that pioglitazone, a thiazolidinedione capable of binding and activating PPARγ^37^, would increase the expression of *Abca1* and *Abcg1* in *Csf2*−/− macrophages and reduce the PAP disease severity after oral administration in *Csf2rb*−/− mice.

**Figure 5:**
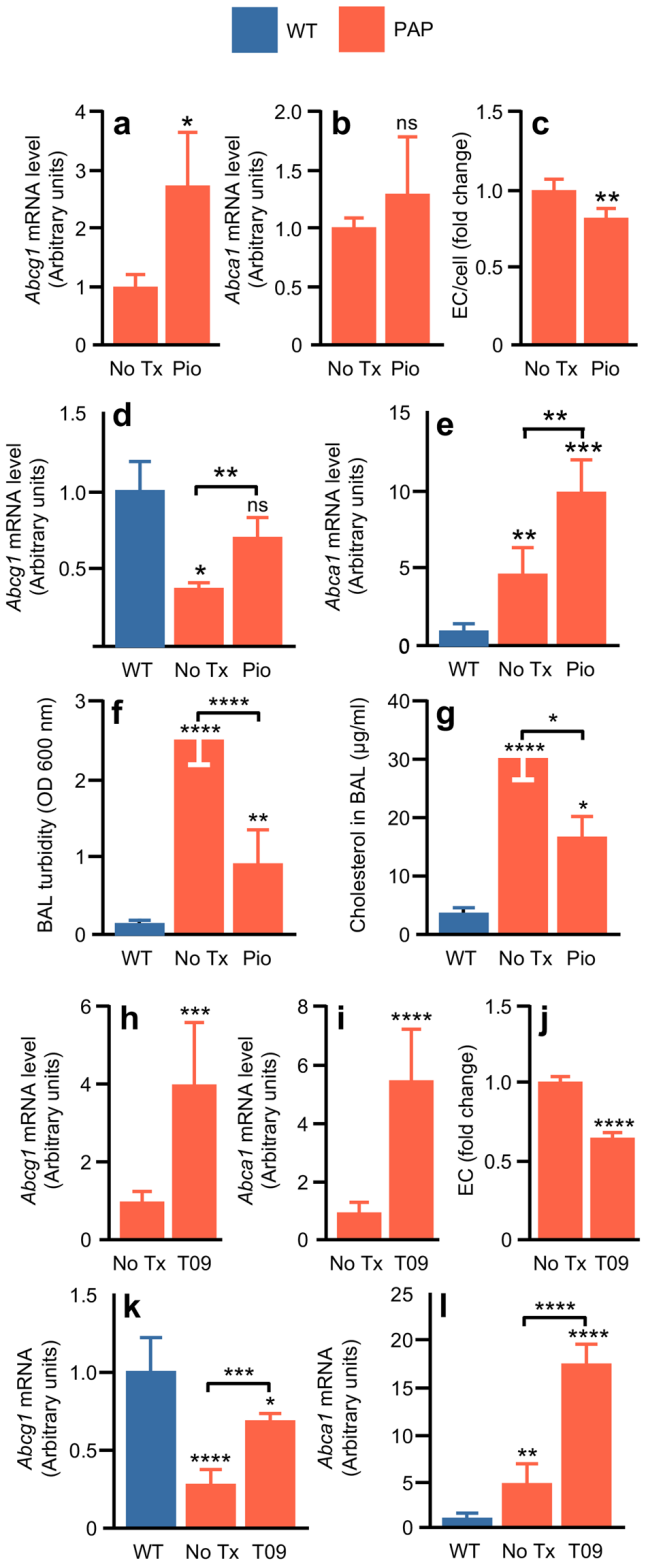
Pharmacologic targeting of cholesterol homeostasis in PAP. (**a**,**b**) mRNA levels of Abcag1 or Abca1 in cultured mouse BMD macrophages after incubation for 24 hours without (No Tx) or with pioglitazone (Pio) determined by RT-PCR. (**c**) BMD macrophages from PAP mice cultured for 24 hours without (No Tx) or with pioglitazone (Pio) were then exposed to PAP-patient derived surfactant (PAP-S) for 24 hours and the amount of esterified cholesterol (EC) per cell was measured. (**d**,**e**) mRNA levels of Abcg1 and Abca1 in primary alveolar macrophages from PAP mice receiving low dose pioglitazone (Pio) for six weeks, or in age-matched, untreated WT or PAP mice (No Tx) (n = 4 mice per condition). (**f**) BAL turbidity and BAL cholesterol levels (n = 8 mice per condition) (**g**) in PAP mice receiving low dose pioglitazone (Pio) for six weeks, or in age-matched, untreated WT or PAP mice (No Tx). (**h**,**i**) Relative levels of *Abcg1* (**h**) or *Abca1* (**i**) mRNA in BMD macrophages from PAP mice after culture for 24 hours with T0901317 (T09). (**j**) BMD macrophages from PAP mice cultured for 24 hours with T09 or without T09 were then exposed to PAP-S for 24 hours and the amount of esterified cholesterol per cell was measured. (**k**,**l**) *In vivo* T0901317 study. T09 was administered by oral gavage once daily for 7 days at a dose of 10 mg/kg/BW/Day to PAP mice. Relative levels of mRNA for *Abca1* (**k**) or *Abcg1* (**l**) in alveolar macrophages from PAP mice after oral T09 or from untreated, age-matched WT or PAP mice (n = 6 mice per condition). Data are mean ± SD for 4–8 mice/group (4F, 4 M). *P < 0.05, **P < 0.01, ***P < 0.001, ****P < 0.0001.

Exposure of alveolar macrophages from PAP mice to pioglitazone *in vitro* markedly increased *Abcg1* mRNA compared to untreated controls but did not significantly alter *Abca1* mRNA levels (Fig. 5a,b). Furthermore, exposure of macrophages from PAP mice to pioglitazone reduced cholesterol ester accumulation following exposure to PAP-surfactant *in vitro* (Fig. 5c).

Since pioglitazone is available as an FDA-approved, commercially available, oral medication, we evaluated its efficacy as therapy of PAP in mice. Pioglitazone was administered to *Csf2rb*−/− mice orally by inclusion in the food to achieve a low-moderate dose (20 mg/kg/day) for six weeks. Pioglitazone treatment significantly increased levels of both *Abcg1* and *Abca1* mRNA in alveolar macrophages compared to untreated, age-matched controls (Fig. 5d,e). Quantitative assessment of PPARγ mRNA levels in alveolar macrophages showed no significant differences between treated and untreated groups (Data not shown). Pioglitazone also significantly reduced BAL turbidity (Fig. 5f) and cholesterol level in *Csf2rb*−/− mice compared to untreated, age-matched controls (Fig. 5g). Oral pioglitazone had no apparent adverse effects in *Csf2rb*−/− mice (not shown). These results identify PPARγ as a molecular target for regulating cholesterol homeostasis in the lungs and support the feasibility of pioglitazone as a novel pharmacologic therapy of PAP.

Since LXRα also regulates ABCA1 and ABCG1 expression^38^, we evaluated it as a second molecular target. *In vitro* exposure of alveolar macrophages from PAP mice to T0901317, an LXR agonist previously evaluated as a potential cholesterol-related therapy of atherosclerosis^39^, caused an increase in *Abcg1* and *Abca1* mRNA (Fig. 5h,i) consistent with known effects of this transcriptional regulator^40, 41^. Moreover, *in vitro* exposure of BMD macrophages from *Csf2*−/− mice to T0901317 reduced cholesterol ester accumulation following exposure to PAP-surfactant (Fig. 5j). Oral administration of T0901317 to *Csf2rb*−/− mice (10 mg/kg/day, 7 days) significantly increased *Abcg1* and *Abca1* mRNA levels in alveolar macrophages compared to untreated, age-matched controls (Fig. 5k,l). These pre-clinical results support the potential feasibility of LXR as a cholesterol-related molecular target for pharmacotherapy of PAP.

## Discussion

This study identified mechanisms by which GM-CSF regulates lipid homeostasis in macrophages, which is critical to surfactant homeostasis, alveolar stability and lung function, and by which disruption of GM-CSF signaling causes PAP syndrome. GM-CSF stimulated cholesterol clearance in macrophages in a constitutive, reversible, and concentration-dependent manner. Loss of GM-CSF stimulation caused macrophage foam-cell formation after surfactant exposure and altered lung surfactant composition by increasing the relative proportion of cholesterol. PPARγ and LXRα were identified as molecular targets for cholesterol-related pharmacotherapy of PAP.

**Figure 6:**
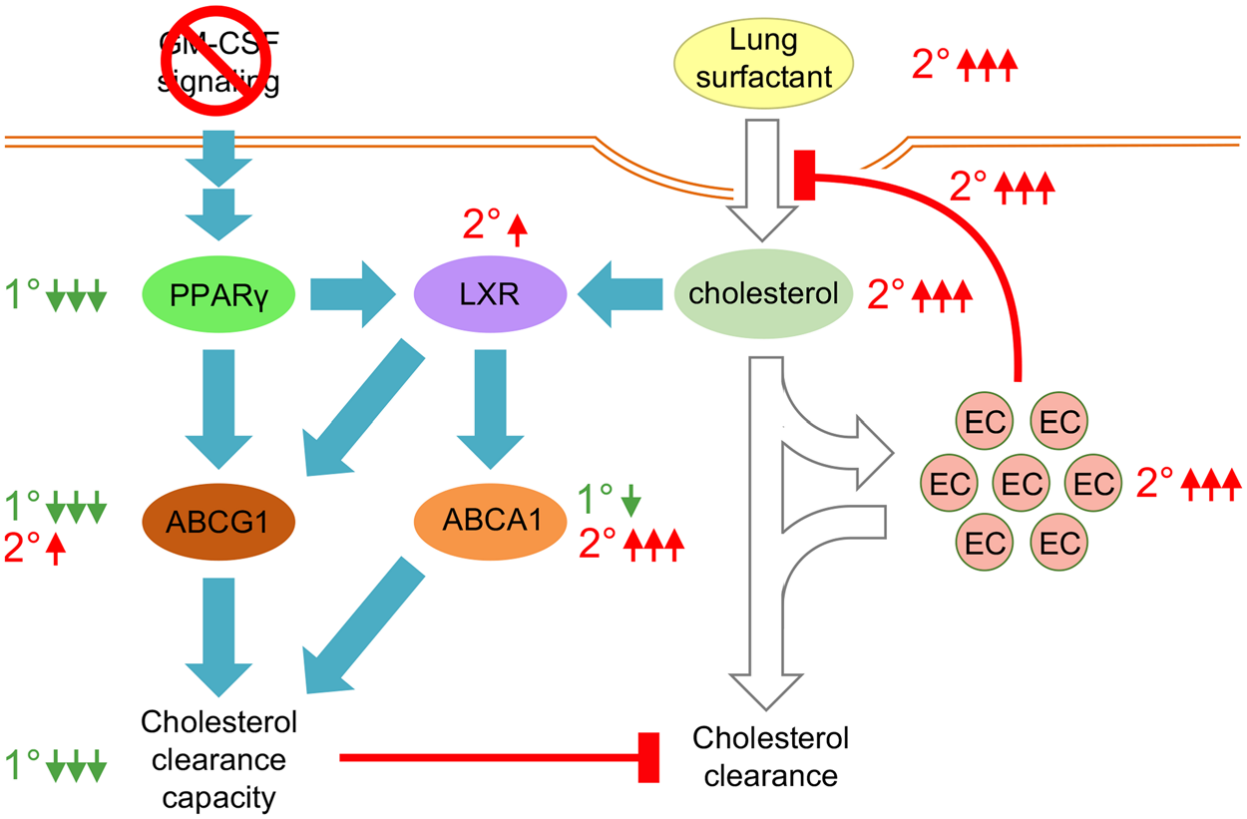
Proposed mechanism by which GM-CSF regulates cholesterol homeostasis in alveolar macrophages and its disruption causes PAP. Shown are signaling pathways (blue arrows), the primary effects of GM-CSF signaling disruption (green numbers/arrows) and the secondary consequences of exposure to cholesterol-containing surfactant (red numbers/arrows).

Disruption of GM-CSF signaling caused a complex pattern of macrophage defects including primary abnormalities attributable directly to the loss of GM-CSF stimulation and secondary abnormalities resulting from subsequent exposure to surfactant (Fig. 6). Primary defects, which were not limited to lung macrophages, included suppression of the GM-CSF > PU.1 > PPARγ signaling axis, reduced expression of ABCG1 and other cholesterol clearance-related genes and reduced cholesterol clearance capacity but did not result in an inability to catabolize surfactant phospholipids. Secondary abnormalities, which occurred only after exposure to cholesterol-containing surfactant, included foam-cell formation and reduction in surfactant uptake and clearance, and increased expression of cholesterol-stimulated, LXR-target genes such as ABCA1^40^. Phagocytic function was reduced primarily by the absence of GM-CSF and secondarily by exposure to cholesterol-containing surfactant in both normal and PAP macrophages. The mechanism identified explains Golde’s ‘stuffed macrophage’ hypothesis of PAP pathogenesis^42^ and explains why cytopathologic abnormalities occur in alveolar but not other tissue macrophages and also their pattern of ABCA1 and ABCG1 expression^23^. The accumulation of cholesterol in alveolar macrophages has implications for the increased infection risk in PAP^43^ since facultative intracellular organisms such as mycobacteria, particularly *M*. *tuberculosis*, preferentially invade and replicate in alveolar macrophages, can themselves induce foam-cell formation^44^ and utilize cholesterol as a carbon source^45, 46^, which may promote persistence^45^.

The observation of an increased ratio of cholesterol to phospholipid in PAP challenges the concept that PAP arises from accumulation of surfactant of essentially normal lipid composition^15, 21^. Importantly, the latter view derives largely from studies restricted to evaluating the polar lipid fraction of surfactant^20^ rather than total surfactant lipids as in our study. Our findings are supported by a report focused to surfactant proteins in PAP that incidentally noted an increase in cholesterol and another reporting an increase in cholesterol in alveolar macrophages in PAP^23, 47, 48^. Our results also agree with a previous report indicating surfactant phospholipids and cholesterol are metabolized in macrophages via distinct pathways^49^ and extend it by showing that a reduction in cholesterol clearance results in formation of foam-cells with secondarily impaired uptake and clearance of surfactant phospholipids.

These results have clinical implications for pharmacotherapy of autoimmune PAP, hereditary PAP, other surfactant-related lung diseases, and possibly other diseases associated with macrophage foam-cell formation. The observation that GM-CSF constitutively regulates cholesterol clearance relates to the timing of administration of inhaled GM-CSF as pharmacotherapy of PAP, which is currently prescribed ‘off-label’ once daily on alternating weeks^50^. The observation that pioglitazone increased Abcg1 expression in macrophages, reduced cholesterol accumulation, and reduced disease severity in PAP mice supports the feasibility of its use as pharmacotherapy of PAP in humans. It is interesting that pioglitazone was recently shown to reduce the risk of stroke or myocardial infarction in patients with insulin resistance^51^. Our results have mechanistic implications for this clinical observation related to potential for improving cholesterol export by foam-cells within atherosclerotic plaques. That T0901317 had similar effects on Abcg1 and Abca1 expression and cholesterol clearance, suggesting that targeting LXR activity may be another feasible pharmacotherapeutic approach. Importantly, this study has already supported clinical translation through initiation of a first-in-human clinical trial of oral pioglitazone therapy in patients with autoimmune PAP.

The limitations of our study include that it did not precisely define the molecular mechanism by which disruption of GM-CSF signaling impairs cholesterol clearance or the increased cholesterol to phospholipid ratio observed in PAP. A possible limitation of this study may be the inability to account for the mechanical compression and expansion which occurs in in the lung, which possibly affects surfactant uptake and clearance in alveolar type II cells^52^, however, the role of mechanical stretch on regulating surfactant uptake and clearance in alveolar macrophages is still unknown. Additionally, results did not establish a dose-response relationship or time to optimal treatment effect for pioglitazone therapy of murine PAP or establish the therapeutic efficacy of T0901317 *in vivo*. Thus, additional studies will be needed to further define the mechanisms identified here and continue pharmacotherapeutic translation.

## Methods

### Ethical Approval

All methods, procedures and experiments in this study were performed in accordance with relevant guidelines and regulations. A total 154 mice were used in this study.

### Bronchoalveolar lavage and alveolar macrophage collection in mice

GM-CSF (*Csf2*) knockout (*Csf2* ^**−/−**^) mice^5^ and *Csf2rb* gene-deficient (*Csf2rb* ^**−/−**^)^14^ were reported previously (referred to mouse PAP). C57Bl/6 mice (referred to as wild type or WT mice) were purchased from Charles River. All mice were bred, housed, and studied in the Cincinnati Children’s Research Foundation Vivarium using protocols approved by the Institutional Animal Care and Use Committee. Epithelial lining fluid and non-adherent cells were collected from indicated mice by bronchoalveolar lavage (BAL) using five 1 ml aliquots of PBS plus 0.5 mM EDTA^4^. The 1 ml aliquots were pooled and the recovered volumes recorded. The supernatant was removed and the cellular pellets were resuspended in the culture media for isolation of alveolar macrophages by adherence to tissue culture plastic.

### Oil red O staining

Cells were stained with Oil Red O staining using the Oil red O staining kit (Poly Scientific R&D Corporation) according to the following protocol. Briefly, cells were fixed with 4% PFA and washed twice with distilled water. Cells were placed in absolute propylene glycol for 5 minutes. Propylene glycol was removed and cells stained in a 0.5% oil red o solution in propylene glycol for 30 minutes. Cells were rinsed in an 85% propylene glycol solution for 5 minutes and washed twice with distilled water followed by a hematoxylin counterstain for 2 minutes. Cells were mounted with an aqueous mounting medium such as glycerin jelly and evaluated by light microscopy.

### Tri-one dimensional thin layer chromatography (TOD-TLC) analysis

Alveolar macrophages were isolated from BALF based on adherence to tissue culture plastic as described above. Cellular lipids analysis was performed on AMs isolated from individual WT mice and two pooled mice per sample for *Csf2* ^**−/−**^ mice. Cells were repeatedly washed with PBS to remove extracellular surfactant and then 100% isopropanol was added to the tissue culture wells, 2mls for a 6-well plate. Cellular lipids were extracted for 2 hrs at room temperature or overnight at 4 °C. The isopropanol was then transferred into glass tubes and half the volume of new isopropanol was added back into the tissue culture plate for 30 minutes to recover any remaining sample and combined with the original volume. Lipid samples were then evaporated using a stream of nitrogen and a water bath set to 52°, once evaporated the samples were reconstituted in chloroform. Cellular lipid samples were then loaded onto high performance thin layer chromatography plates pre-coated with silica gel 60 (Fisher). Plates were prewashed with chloroform and methanol to remove any contaminants and dried overnight at 120 °C. Plates were developed in a solvent system modified from White *et al*.^53^. Briefly, plates were first developed in a Solvent mixture of chloroform, ethanol, triethylamine, and water (30:35:35:6) up to 7 centimeters of a ten centimeter plate. Plates are removed from the chamber, dried, and placed in a second solvent of hexane and diethylether (90:10) up to nine centimeters of a ten centimeter plate. Plates are again removed from the chamber, dried, and then placed in the final solvent of pure hexane and run to the top of the plate. Bands are visualized by spraying with a 0.05% solution of primuline in acetone and water (80:20) and detected as ultraviolet spots at 366 nm on a Typhoon 9500 molecular imager^53^.

### Tissue macrophage differentiation and culture

All cells were maintained in the culture medium of Dulbecco’s modified eagle’s medium (DMEM) (Life Technologies) plus 10% FBS, 50 U ml^−1^ penicillin, and 50 µg ml^−1^ streptomycin. Bone marrow cells were obtained from 6–8 week old mice by flushing the tibias and femurs with the culture media described above. Mononuclear cells were isolated by centrifugation over a Ficoll-Paque (GE Healthcare Life Sciences) gradient at room temperature for 30 minutes. The buffy coat was washed in PBS and the cellular pellet resuspended in the culture medium with M-CSF (R&D Systems) (10 ng/ml) plus or minus GM-CSF (R&D Systems) as dictated per experimental requirements. Cells were cultured in a 10 cm dish overnight at 37 °C and the next day non-adherent cells were recovered and transferred to a new dish and cultured under the same conditions for an additional 24hrs. At this stage non-adherent cells were discarded and adherent cells cultured for an additional five days to allow differentiation of bone marrow derived (BMD) macrophages. Peritoneal macrophages were isolated from the peritoneal without chemical solicitation by washing with 8 ml of PBS plus 0.5 mM EDTA. WT peritoneal macrophages were cultured *in vitro* in culture medium with M-CSF (10 ng/ml) and GM-CSF (10 ng/ml), and *Csf2* ^**−/−**^ peritoneal macrophages were cultured in culture medium with M-CSF alone.

### Cellular Lipid Analysis and Protein Concentration

To collect cellular lipids, 100% isopropanol was added to the tissue culture wells. Cellular lipids were extracted for 2 hrs at room temperature or overnight at 4 °C. The isopropanol was then transferred into glass tubes and half the volume of new isopropanol was added back into the tissue culture plate for 30 minutes to recover any remaining sample and combined with the original volume. Following removal of isopropanol, a Pierce BCA (bicinchoninic acid assay) protein assay (Thermo Fisher Scientific) was performed on the tissue culture wells to determine the cellular protein concentration.

### Bronchoalveolar lavage fluid analysis

#### Turbidity

The turbidity of the fluid was measured as previously described^4, 54^. Briefly, 250 uls of the BAL were diluted into 750 uls of PBS and the optical density measured at a wavelength of 600 nm and multiplied by the dilution factor.

#### Lipid Extraction from BAL fluid

A chloroform-methanol extraction was employed to analyze lipid species. Briefly, 1 ml of BAL was diluted with 1 ml of DPBS, subsequently 2 ml of 100% methanol, and 4 ml of 100% chloroform were added. This was mixed and then centrifuged at 4 °C @ 1000 rpm. The lower phase containing the extracted lipids was transferred to a new glass tube.

#### Cholesterol levels

Total and free cholesterol levels were measured by the amplex red cholesterol assay (Life Technologies) according to the manufacturer’s protocol. Esterified cholesterol was then calculated by subtracting free cholesterol from the total value.

#### Phospholipid levels

Aliquots of the BAL were taken as pre-spun samples and total BAL lipids were extracted using chloroform and methanol. Total phosphate and saturated phosphatidylcholine were measured as previously reported^55, 56^.

#### *In vitro* surfactant challenge assay

BMD macrophages or peritoneal macrophages were seeded at 4 × 10^5^ cells per well of a 12 well tissue culture plate and allowed to adhere with fresh media and cytokines overnight. Cells were then challenged for 24 hrs with pulmonary surfactant isolated from a hereditary PAP patient (PAP-S). After the challenge cells were gently washed with PBS to remove exogenous PAP-S and cellular lipids were then extracted and analyzed by the amplex red cholesterol assay.

#### Liposome and fluorescently labeled surfactant preparation

Lipids, cholesterol or rhodamine-dipalmitoyl phosphatidylethanolamine (R-DPPE), were purchased from Avanti Polar Lipids as organic solutions in chloroform. Survanta lipids (Sv-S) (Abbvie) were extracted using chloroform and methanol and to the extracted chloroform phase the additional lipids, cholesterol or R-DPPE, were combined in a glass tube and vortexed. Solvents were evaporated using a stream of nitrogen and a water bath at 52 °C. PBS prewarmed to 37 °C was added to the dried lipids and placed in a 45 °C water bath for 15 minutes. Samples were removed and immediately vortexed, this process was repeat twice. Using a bath sonicator the samples were briefly sonicated and transferred to a glass vial with a Teflon lined cap before use. For experiments involving fluorescently labeled surfactant Sv-S was chloroform methanol extracted and fluorescently labeled lipids were added to the extracted Survanta samples in chloroform and vortexed vigorously. Samples were dried and the above listed procedure used to prepare liposomes. Cells were challenged at a final concentration of 250 ug/ml of total lipid with the fluorescent label present at a 1:10 ratio. Uptake experiments were performed for 30 minutes at 37 °C as previously reported^57^. Cells were collected, fixed, and analyzed by flow cytometry. Kinetic experiments were performed by loading cells for 24 hrs with fluorescently labeled surfactant. Extracellular surfactant was removed by gentle washing with PBS. Cells were collected for an immediate loaded time point or 3 hr, 8 hr, and 24 hrs after washing.

#### Cholesterol reconstitution studies

Free cholesterol was added to chloroform and methanol extracted Sv-S lipids in chloroform and liposomes were prepared as previously described. Sv-S or Sv-S plus cholesterol at 250 ug/ml was then used as a lipid source to challenge macrophages *in vitro* for 24 hrs. After the challenge cells were gently washed with PBS and cellular lipids were extracted or RNA isolated and analyzed as previously described. For examining cholesterol dependent effects on surfactant uptake macrophages were pre-challenged for 24 hrs with Sv-S or Sv-S plus cholesterol. Following the pre-challenge period cells were washed gently with PBS to remove exogenous lipid and challenged for an additional 30 minutes with R-DPPE labeled Sv-S at 37 °C. Cells were washed with PBS and immediately fixed with 4% PFA. Surfactant uptake was measured by flow cytometry detection of R-DPPE inside the cells.

#### Kinetics of cholesterol clearance

For kinetic experiments *Csf2* ^**−/−**^ BMD macrophages were differentiated and maintained throughout the course of the study in M-CSF while WT BMD macrophages received both M-CSF and GM-CSF. On the 7^th^ day cells were seeded into 12 well plates and allowed to adhere overnight in fresh media with cytokines. Cells were then challenged with PAP-S for 24 hrs. Cells were collected immediately, as a loaded control, and 3, 8, 24, 48, and 72 hrs after washing to remove PAP-S to track changes in cholesterol homeostasis overtime. Cellular lipids were extracted, normalized to total protein, and analyzed.

#### Phagocytosis assay

The phagocytic capacity of WT and *Csf2rb*−/− BMD macrophages after PAP-S challenge was evaluated using opsonized Nile red beads (Spherotech) as described previously^58^. The mean fluorescence intensity of the phagocytosed beads was measured using FACS Canto 1 (BD biosciences) and histogram overlays was done using FlowJo software.

### GM-CSF plasticity studies

#### GM-CSF withdrawal

*Csf2* ^**−/−**^ BMD macrophages were differentiated and maintained in media containing M-CSF and GM-CSF. After 7 days of differentiation cells were split into 12 well tissue culture plates and allowed to adhere overnight in GM-CSF containing media. The following day the media was changed to M-CSF only and cells were challenged with PAP-S for 24 hrs immediately or 24, 48, and 72 hours after GM-CSF withdrawal. Cellular lipids were extracted, normalized to total protein, and analyzed. *Csf2* ^**−/−**^ BMD macrophages were used to ensure elimination of any potential autologous GM-CSF expression-related stimulation.

#### Multi-Day GM-CSF stimulation

*Csf2* ^**−/−**^ BMD macrophages were generated in media containing M-CSF. On the 7^th^ day cells were stimulated for 0, 24, 48, or 72 hrs with 10 ng/ml of GM-CSF before 24 hr surfactant challenge with PAP-S. Cellular lipids were extracted, normalized to total protein, and analyzed.

#### GM-CSF dose response

*Csf2* ^**−/−**^ BMD macrophages were generated in media containing M-CSF. On the 7^th^ day cells were stimulated by a dose response of GM-CSF (0, 0.1,1,10, and 100 ng/ml) for 24 hrs before surfactant challenge with PAP-S. Cellular lipids were extracted, normalized to total protein, and analyzed.

#### RNA isolation and gene expression analysis

Total RNA was isolated using Trizol (Life Technologies) and was converted to cDNA using the Invitrogen Superscript III first strand synthesis kit (Life Technologies) according to the manufacturer’s protocol. Standard quantitative RT-PCR (qRT-PCR) was performed as previously described^10^ on an Applied Biosystems 7300 Real-Time PCR System (Life Technologies) to measure transcript abundance using TaqMan® oligonucleotide primer sets (all from Life Technologies). Expression of target genes was normalized to the expression of 18s RNA.

### Molecular Therapy Experiments

#### *In vitro* mouse PPARγ & LXR stimulation Studies

BMD macrophages were prepared as previously described in the presence of M-CSF (10 ng/ml). On the 7^th^ day macrophages were stimulated for 24 hrs with M-CSF (10 ng/ml), GM-CSF (10 ng/ml), Pioglitazone (10 µm) or T0901317 (1 µm from Tocris). Macrophages were then used for an *in vitro* surfactant challenge assay or gene expression analysis by qRT-PCR as described above.

#### *In vivo* PPARγ stimulation studies

Pioglitazone was incorporated into standard rodent chow at a dose expected to deliver 20 mg/kg BW/Day. BAL turbidity and alveolar macrophage gene expressions were measured as described above. GM-CSF levels and total cholesterol levels in the BAL were measured in the supernatant of fluid after centrifuge at 283 g for 10 minutes at 4 °C by enzyme-linked immunosorbent assay (ELISA) (R&D systems) and Amplex Red Cholesterol assay respectively as described above.

#### *In vivo* LXR Stimulation Studies

Mice were administered T0901317 by oral gavage once daily for 7 days at a dose of 10 mg/kg/BW/Day using propylene glycol and tween 80 as a vehicle. On the 7^th^ day alveolar macrophages were collected by bronchoalveolar lavage. Following brief adherence to tissue culture plastic to remove BAL debris, alveolar macrophages were then used for RNA isolation and gene expression analysis by qRT-PCR as described above.

## Electronic supplementary material

Supplementary Data
