# Supplementary material for: Targeting cholesterol homeostasis in lung diseases
Source: Sci Rep. 2017 Aug 31;7:10211. doi: 10.1038/s41598-017-10879-w (PMC5579240; doi:10.1038/s41598-017-10879-w)
Supplement: Supplementary file 1 — Supplementary Data [file 41598_2017_10879_MOESM1_ESM.pdf]

Targeting cholesterol homeostasis in lung diseases

Anthony Sallese,[1,2,3] Takuji Suzuki,[1,2] Cormac McCarthy,[1,2,4,5] James Bridges,[2] Alyssa Filuta,[2] Paritha Arumugam,[1,2] Kenjiro Shima,[1,2] Yan Ma, [1,2] Matthew Wessendarp,[1,2] Diane Black,[1,2] Claudia Chalk,[1,2] Brenna Carey,[1,2] Bruce C. Trapnell,[1,2,4,5]

1 Translational Pulmonary Science Center, Children's Hospital Medical Center, Cincinnati, OH, USA;

2 Division of Pulmonary Biology, Children's Hospital Medical Center, Cincinnati, OH, USA;

3 Graduate Program in Pathobiology and Molecular Medicine, University of Cincinnati College of

Medicine, Cincinnati, OH, USA;

4 Division of Pulmonary Medicine, Children's Hospital Medical Center, Cincinnati, OH, USA;

5 Division of Pulmonary, Critical Care, and Sleep Medicine, University of Cincinnati College of Medicine, Cincinnati, OH, USA;

Supplementary Material.

SUPPLEMENTARY FIGURE LEGEND

Supplementary Figure 1| Expression of Cholesterol Regulatory Genes in PAP macrophages.

a-d, Relative levels of mRNA for *Lxrα* (**a**) *Acat1* (**b**), *Nceh1* (**c**) and *Lipa* (**d**) in alveolar macrophages of WT and PAP mice measured by quantitative real time polymerase chain reaction (qRT-PCR) analysis. **e-h**, BMD macrophages from WT or PAP mice were exposed to PAP-S. After 24 hours, cellular mRNA levels of *Lxrα* (**e**) *Acat1* (**f**), *Nceh1* (**g**) and *Lipa* (**h**) were measured by RT-PCR analysis. Data represent the mean ± SD of 3 separate determinations per condition. *P<0.05, **P<0.01, ***P<0.001, ****P<0.0001.

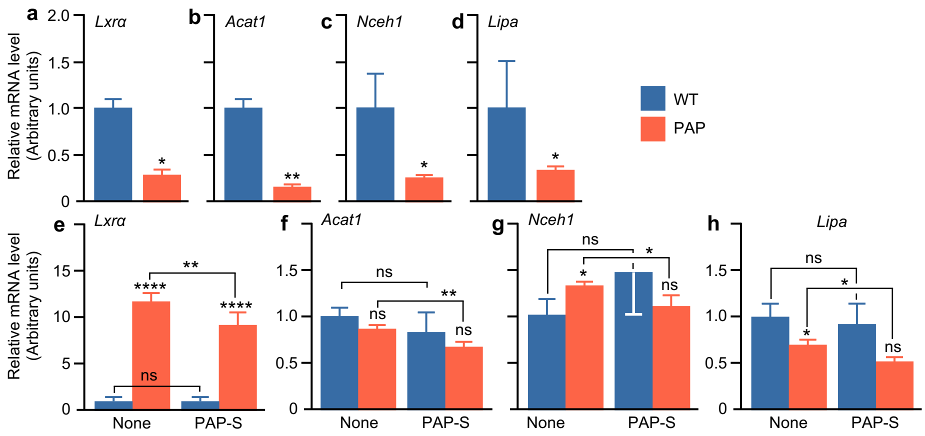

a
b
c
d
Lxrα
Acat1
Nceh1
Lipa
Relative mRNA level (Arbitrary units)
WT
PAP
e
f
g
h
None
PAP-S
ns
